# An mHealth Intervention (ReZone) to Help Young People Self-Manage Overwhelming Feelings: Cluster-Randomized Controlled Trial

**DOI:** 10.2196/14223

**Published:** 2020-07-27

**Authors:** Chloe Edridge, Miranda Wolpert, Jessica Deighton, Julian Edbrooke-Childs

**Affiliations:** 1 Clinical, Educational and Health Psychology University College London London United Kingdom; 2 Anna Freud National Centre for Children and Families London United Kingdom

**Keywords:** cluster trial, behavioral difficulties, schools, mHealth, digital, mental health

## Abstract

**Background:**

Mental health difficulties in young people are increasing, and there is a need for evidence on the effectiveness of digital interventions to increase opportunities for supporting mental health in young people. Such studies are complicated due to issues of implementation and adoption, outcome measurement, and appropriate study designs.

**Objective:**

The objective of this study was to examine the effectiveness of an mHealth intervention (ReZone) in reducing mental health difficulties in young people.

**Methods:**

The cluster-randomized controlled trial enrolled 409 participants aged 10-15 years, and classes were allocated to ReZone or management as usual. Self-reported questionnaires were completed at baseline and 3-month follow-up.

**Results:**

There were no significant differences between the ReZone condition and management as usual in the self-reported outcome measures. However, there were 3467 usage sessions, which corresponds to 16.9 times per student (total of 205 students) in classes allocated to ReZone.

**Conclusions:**

It is essential to publish studies that do not show significant differences, as these findings can still contribute to the literature, help in learning, and inform the direction of future work. The results reported in this paper could be due to a range of reasons, including whether ReZone has the scope to impact change or limitations related to the setting, context, and appropriateness of an RCT. The findings of this study suggest that ReZone was implemented and adopted.

**International Registered Report Identifier (IRRID):**

RR2-10.2196/resprot.7019

## Introduction

Mental health difficulties are increasing among young people. Emotional disorders are the most prevalent and show the most significant increase over time and with age [1,2]. Recent figures show that 1 in 8 (12.8%) people aged 5-19 years had at least one mental disorder when assessed [3]. Mobile health (or mHealth) interventions have the potential to provide mental health support at scale, and there is a need for evidence as to the effectiveness of digital interventions for supporting mental health in young people [4]. However, non-adoption and implementation issues present substantial barriers to effectively doing so [5]. Additionally, questions about whether randomized controlled trials are always the most appropriate method of evaluation need to be considered in the context of practicality and feasibility factors [6-8].

The authors have co-designed an mHealth intervention, ReZone, with young people, parents/carers, and teachers, based on cognitive behavioral therapy (CBT), mindfulness, and attention bias modification training (ABMT). This short paper will report the findings from a cluster randomized controlled trial (RCT) to test the hypothesis that young people in classes receiving ReZone have fewer behavioral difficulties than young people in classes receiving management as usual. Full details on the methods and development are reported in the protocol [9] and implementation paper [10].

## Methods

### Participants and Procedure

A total of 49 classes were recruited (27 mainstream, 22 alternative provision), resulting in a sample of 409 students (mean age 10.9, SD 1.44 years; 56% male; Table 1). Schools were recruited via newsletters and events published in our Schools in Mind network. Participants were enrolled from 12 mainstream primary schools (307 students) and 11 secondary alternative provision schools (103 students). A total of 66 people were lost to follow-up, resulting in an attrition rate of 16%. All students (aged 10-15 years) in schools taking part in the project were eligible to participate. Classes were randomized using a true random number generator in blocks of two by an independent trials unit, to management as usual or ReZone stratified by school type (alternative provision vs. mainstream) after classes were recruited and baseline measures assessed. Ethical approval was received from University College London (UCL) Research Ethics Committee (number: 7969/001). The present research is reported in line with CONSORT guidelines [11].

**Table 1:** Demographic characteristics and evaluation statistics.

Demographics		Overall	Mainstream primary			Alternative provision		
			Control	Intervention	*P* value	Control	Intervention	*P* value
Age (years), mean (SD)		10.9 (1.44)	10.25 (0.43)	10.28 (0.45)	.53	13.6 (1.48)	12.3 (1.45)	<.001
Male, n (%)		227 (56)	69 (43.9)	75 (50.3)	.22	38 (80.9)	45 (80.4)	.90
**Ethnicity, n (%)**					.74			.90
	White	247 (60)	98 (62.4)	82 (55.0)		31 (66.0)	36 (64.3)	
	Mixed-race	29 (7)	N/A^a^	6 (4.0)		7 (14.9)	9 (16.1)	
	Asian	78 (19)	34 (21.7)	40 (26.9)		3 (6.4)	1 (1.8)	
	Black	40 (10)	11 (7.0)	15 (10.1)		5 (10.6)	9 (16.1)	
	Other	15 (4)	5 (3.2)	5 (3.4)		7 (14.9)	1 (1.8)	
Disabled, n (%)		28 (3.4)	2 (1.3)	2 (1.3)	.99	6 (13.0)	4 (7.3)	.28
Alternate provision schools, n (%)		103 (25)	N/A	N/A	N/A	N/A	N/A	N/A
English first language, n (%)		352 (87)	N/A	N/A	N/A	N/A	N/A	N/A
**Usage, n**								
	Happy faces	1169	N/A	N/A	N/A	N/A	N/A	N/A
	Breathing	811	N/A	N/A	N/A	N/A	N/A	N/A
	Game	752	N/A	N/A	N/A	N/A	N/A	N/A
	Stress bucket	404	N/A	N/A	N/A	N/A	N/A	N/A
	Art	225	N/A	N/A	N/A	N/A	N/A	N/A
	Time out	106	N/A	N/A	N/A	N/A	N/A	N/A

^a^N/A: not applicable.

### Intervention: ReZone App

Full details of the ReZone intervention are described in the protocol [9]. The intervention primarily aims to reduce behavioral difficulties. It is available as a web-based smartphone and tablet application flexibly designed to be used in groups or individually, with or without adult support depending on the user’s capability and confidence. This flexibility also extends to the order and frequency in which elements of the tool are used. However, it is required to have an initial guided session or tutorial with an adult. This flexible approach to implementation aimed to fit within a variety of schools and their differing student characteristics, priorities, and timetables.

The primary features of ReZone (Textbox 1) are presented below.

**The primary features of ReZone.**

**Stress bucket.** The stress bucket lets the user add any stressors that they are experiencing to a bucket. They are then able to introduce activities that help them cope with each stressor. They can see the water in the bucket rise and fall as they add and relieve stressors. If the bucket reaches 50 stress points, it will overflow.

**Timeout.** Timeout asks the user to think through a time when they have felt stressed, angry, or upset. The user works through the events leading up to feeling this way and what happened afterward. The user can also think through what they could have done differently, thus guiding them to develop a behavioral plan. The timeout visualization is a rocket, and each thought process creates an exhaust cloud.

**Chill out.** Chill out uses breathing to help the user calm down and relax. Each chill out activity is based around an object or animal (ie, rabbit, jellyfish, ball, or square), using breathing in different ways.

**Art therapy.** The user can choose between a castle, dinosaur, fish, goat, heart, helicopter, unicorn, rocket, footballer, sea, or turtle to color in. There is a range of colors and tools to complete the drawing.

**Happy faces.** The user is given 30 seconds to find as many happy faces as they can, amongst other faces depicting negative emotions.

**Game*.*** In the game Balloon Blast, the user taps the screen to move a balloon up, trying to avoid all the obstacles as hitting one will mean the game is over. The game provides a break or reward for the user in between uses of the other features.

### Measures

All measures were self-reported by young people and administered in school by researchers or school staff in paper form. Baseline demographic data were collected and are reported in Table 1. Emotional (α=.78) and behavioral difficulties (α=.81) were measured using the 16-item Me and My School (M&MS) questionnaire [12]. Mental wellbeing was measured using the 7-item Short Warwick-Edinburgh Mental Wellbeing Scale (α=.83) [13]. Empowerment was measured using the 6-item “self” subscale of the Youth Empowerment Scale-Mental Health (α=.73) [14]. Health-related quality of life was measured using the 6-item (with global health visual analog scale) EQ-5D-Y (α=.63) [15].

### Intervention Usage

Intervention usage data were collected in ReZone and exported to the data analysis software. There were 3467 usage sessions, which corresponds to 16.9 times per each of the 205 students in classes allocated to ReZone. The game (n=752), breathing exercises (n=811), and happy faces (n=1169) were used the most; stress bucket (n=404), art (n=225), and time out (n=106) were used the least. Based on teacher consultations, which are discussed elsewhere [10], usage methods and frequency varied across schools and students due to a variety of factors, including student characteristics, teacher priorities, and school environment.

### Analytic Strategy

Means and standard deviations, for all measures, across conditions at baseline and follow-up, were calculated. Intention-to-treat and per-protocol analyses with four multilevel regressions were tested for each of the outcome variables with time nested within students within classrooms. Per-protocol analyses included only those that had completed follow-up. Model 0 was computed as the null model to examine the change in outcome over time, without predictors. The intraclass correlation coefficients for each measure were 17% (emotional difficulties), 24% (behavioral difficulties), 7% (empowerment), 9% (wellbeing), and 3% (quality of life). We then entered school type (ie, alternative provision vs mainstream) as a fixed effect predictor to Model 1. In Model 2, patient-level grand mean-centered age, ethnicity, gender, and disability were entered as predictors. Demographics were controlled for in the analyses as there were significant differences in gender between groups at baseline within mainstream primary schools and age between groups within alternative provision schools (Table 1). In the Final Model (Model 3), condition (ReZone vs management as usual) was entered as a predictor. The likelihood ratio test was used to compare the fit of Models 1, 2, and 3. For example, Model 1 was significant for behavioral difficulties and therefore compared to Model 2, which was significant. Models 1 and 2 were therefore retained in the final model, with Model 3 (final model) being compared to Model 2 using the likelihood ratio test. Models 1 and 2 were significant for empowerment and wellbeing and therefore retained in the final model. For emotional difficulties and quality of life, only Model 2 was significant and retained in the final Model.

## Results

The means and standard deviations of all study measures at baseline and postintervention are presented in Table 2. The likelihood ratio test for the intention to treat analysis was not significant for any of the final models compared to the previous significant model. There were no significant differences between the ReZone condition and management as usual in emotional difficulties (χ^2^_1_=0, *P*>.05), behavioural difficulties (χ^2^_1_=0.01, *P*>.05), empowerment (χ^2^_1_=0.01, *P*>.05), wellbeing (χ^2^_1_=0.05, *P*>.05), or quality of life (χ^2^_1_=0.3154, *P*>.05). The per-protocol results showed similar patterns.

**Table 2:** Baseline and follow-up mean scores by condition. All values are provided as mean (SD).

	Mainstream primary				Alternative provision			
Outcome	Intervention – T1^a^	Intervention – T2^b^	Control – T1	Control – T2	Intervention – T1	Intervention – T2	Control – T1	Control – T2
Emotional difficulties	5.56 (3.38)	5.18 (3.74)	6.27 (3.38)	5.43 (3.27)	6.08 (3.91)	5.61 (4.12)	5.48 (3.77)	6.08 (4.03)
Behavioral difficulties	3.31 (2.27)	3.28 (2.44)	3.32 (2.24)	3.18 (2.04)	5.45 (2.37)	5.11 (2.34)	5.57 (2.35)	5.82 (2.52)
Empowerment	24.53 (4.57)	25.69 (4.25)	24.65 (4.09)	25.13 (4.03)	22.96 (4.96)	23.61 (5.74)	23.35 (4.02)	23.79 (4.25)
Wellbeing	24.11 (4.78)	24.94 (5.41)	24.41 (5.18)	24.94 (5.06)	21.09 (5.90)	23.61 (5.74)	21.76 (3.98)	21.93 (4.65)
Quality of life	6.13 (1.60)	6.46 (5.86)	6.28 (1.48)	6.29 (6.06)	6.20 (1.24)	4.04 (3.08)	6.30 (1.32)	2.91 (3.14)

^a^T1: Time 1.

^b^T2: Time 2.

## Discussion

This cluster randomized controlled trial examined the effectiveness of ReZone in reducing behavioral and mental health difficulties in young people. The study did not show a significant difference between those using ReZone and management as usual in the reduction of behavioral and emotional difficulties and improving empowerment, wellbeing, and quality of life.

Potential limitations of the study, implementation, and adoption barriers to digital interventions within schools have been discussed in depth [10]. Implementation barriers to new digital interventions in schools include the technology itself, the adopter system, and the organization [5]. The process of embedding ReZone into the school system can sometimes leave minimal time to utilize it successfully. Implementation strategies differed, with varying levels of teacher encouragement and consistency of scheduled ReZone use. Competing teacher priorities, opinions, and seniority of the school advocate could have impacted the time and drive to use the tool [10].

ReZone was used a total of 3467 times throughout the 3-month study period, and current usage data show that ReZone is still being used 10 months poststudy (501 sessions over the last 3 months). Aggregated objective usage data is useful to indicate overall acceptability and use. However, teacher consultations (fully reported in [10]) suggested students either used ReZone frequently or not at all. Analysis of a dose-response effect could be improved by gathering further quantifiable individual school usage information related to implementation.

The usage statistics and teacher consultations indicated happy faces and breathing were the most used activities, while the stress bucket, art, and time out were used the least. Happy faces and breathing can be completed in only 1-2 minutes; they are a fun challenge/game type of activity, and most of the time can be completed without teacher assistance. The stress bucket and time out require more time to work through thought processes, requiring the user to be willing to think and talk about their feelings, needing teacher support (which is not always feasible).

The challenges discussed link to the emerging concept of gamification within digital mental health tools [16,17]. There are some basic elements of gamification within the most popular elements of ReZone. Further work on gamification combined with other crucial processes such as co-design, could help address the broader issue of nonadoption and sustainability, with many users reporting that they stop the use of a health app after only two weeks [18]. This lack of consistency could be particularly applicable when working with young people in such settings as alternative provision schools. Pupil behavior often places higher demands on staff in alternative provision schools than in mainstream schools, and disengagement and loss of concentration are common issues.

Measures used were related to overall mental health, wellbeing, and behavior leading us to question how often a tool such as ReZone would need to be used to effect changes in broad domains and specifically in schools. If tools such as ReZone do not provide large-scale, substantial longer-term changes, we must also ask whether there is any impact on immediate feelings, behavior, and interactions. We did attempt to measure this by asking teachers to complete a few short questions related to how well students refocused and re-engaged after using ReZone. However, due to practical issues such as teacher demand, these were not completed. On reflection, this could have also been something students were asked to complete. It is also possible that effects were delayed, and further long-term data collection, such as one-year post-trial, may have shown differences [19-21].

Due to the complex nature of digital interventions, traditional RCTs may be insufficient to assess efficacy [8]. As this study shows, there are multiple objective and subjective data sources available, including quantitative self-report data, objective usage data, and subjective qualitative data, while digital and school studies present particular challenges in adherence, sustainability, and roll-out [10]. As with psychotherapy research in general and a reliance on self-report measures, participant blinding was not possible, although true and independent randomization was used. Additionally, due to this being a pragmatic trial, there may have been other interventions the school and participants may have been using.

It is essential to publish negative results to avoid publication bias. These studies can still make a substantial contribution to the literature, learning, and informing the direction of future work [22]. The negative results of this study could indicate that ReZone has a limited scope to impact change, that its utility is hampered by issues with implementation and nonadoption, and limitations of mHealth research in a school context. The results may also suggest the limited applicability of the RCT study design for evaluating tools such as ReZone.

## Abbreviations

ABMT: attention bias modification training
CBT: cognitive behavioral therapy
RCT: randomized controlled trial
